# Treatment of HER2-positive metastatic breast cancer with lapatinib and capecitabine in the lapatinib expanded access programme, including efficacy in brain metastases – the UK experience

**DOI:** 10.1038/sj.bjc.6605586

**Published:** 2010-02-23

**Authors:** S Sutherland, S Ashley, D Miles, S Chan, A Wardley, N Davidson, R Bhatti, M Shehata, H Nouras, T Camburn, S R D Johnston

**Affiliations:** 1 Department of Medicine, The Royal Marsden NHS Foundation Trust, Royal Marsden Hospital, Fulham Road, Chelsea and Sutton, London, SW3 6JJ, UK; 2 The Mount Vernon Cancer Centre, Northwood, Middlesex HA6 2RN, UK; 3 Nottingham City Hospital, Hucknall Road, Nottingham NG5 1PB, UK; 4 The Christie Hospital, Manchester, UK; 5 Broomfield Hospital, Chelmsford, Essex, UK

**Keywords:** capecitabine, lapatinib, Her2+ metastatic breast cancer, CNS disease

## Abstract

**Background:**

The global lapatinib expanded access programme provided access to lapatinib combined with capecitabine for women with HER2-positive metastatic breast cancer (MBC) who previously received anthracycline, taxane and trastuzumab.

**Methods:**

Progression-free survival (PFS) and safety data for 356 patients recruited from the United Kingdom are reported. Efficacy was assessed in 162 patients from the five lead centres, including objective tumour response rate (ORR), time to disease progression (TTP) and efficacy in those with central nervous system (CNS) metastases. Correlation of PFS and ORR with previous capecitabine treatment was also documented.

**Results:**

Overall, PFS for the 356 UK patients was 21 weeks (95% CI: 17.6–24.7). In the 162 assessable patients, ORR was 21% (95% CI: 15–27%) and median TTP was 22 weeks (95% CI: 17–27). Efficacy was greater in capecitabine-naive patients (ORR 23 *vs* 16.3%, *P*=0.008). For 34 patients with CNS metastases, ORR was 21% (95% CI: 9–39%), with evidence of improvement in neurological symptoms, and median TTP was 22 weeks (95% CI: 15–28).

**Conclusions:**

Lapatinib combined with capecitabine is an active treatment option for women with refractory HER2-positive MBC, including those with progressive CNS disease.

Despite advances in treatment, breast cancer remains one of the leading causes of cancer-related deaths in women. Approximately 25–30% of patients with breast cancer belong to a subgroup who overexpress the ErbB2 (HER2) oncogene, and this is associated with a higher risk of disease progression and death (Meric *et al*, 2002). Previous studies indicated increased resistance to hormonal and cytotoxic therapies, and a poorer prognosis for patients with tumours overexpressing HER2 (Nicholson *et al*, 2001; Slamon *et al*, 2001). The advent of trastuzumab has radically altered the outlook, such that patients are surviving longer, with the pattern of relapse and systemic disease changing. However, up to a third of patients with HER2-positive metastatic breast cancer (MBC) are now developing central nervous system (CNS) disease, either as a first site of relapse or in addition, despite other sites of systemic disease being well controlled and responding to trastuzumab-based therapy (Bendell *et al*, 2003; Clayton *et al*, 2004). Until recently, once patients developed trastuzumab resistance, further treatment options were limited.

Lapatinib is an orally active small molecule, reversible tyrosine kinase inhibitor (TKI) targeting both HER2 and ErbB1 (EGFR) pathways, and has the potential to be used when trastuzumab resistance develops. In an open-label randomised phase III trial (EGF100151), lapatinib (1250 mg daily) in combination with capecitabine (2000 mg m^–2^ per day days 1–14 every 21 days) significantly improved time to disease progression (TTP) compared with capecitabine alone (2500 mg m^–2^ per day days 1–14, every 21 days). Patients enrolled into EGF100151 had refractory locally advanced or MBC that had progressed after previous treatment including trastuzumab. Interim analysis showed statistically significant superiority of the combination arm in terms of disease progression events (hazard ratio 0.51, 95% CI: 0.35–0.74; *P*<0.001), and the study was stopped early by the independent data and safety monitoring committee (Geyer *et al*, 2006), After completion analysis, the median TTP was 6.2 months for the combination arm compared with 4.3 months with capecitabine alone, with a trend towards improved overall survival. (HR 0.78, 95% CI: 0.55–1.12, *P*=0.177). Interestingly, although the number of patients developing CNS disease was small, fewer patients developed brain metastases as first site of progression on the combination compared with those on capecitabine alone (4 *vs* 13, *P*=0.045) (Cameron *et al*, 2008).

These latter data suggested that lapatinib may help reduce the risk of developing CNS metastases, and that it could be more effective than the monoclonal antibody trastuzumab in treating CNS disease once it occurs. A phase II study with single agent lapatinib 750 mg bid was undertaken in patients with HER2-positive CNS metastases who had shown progression in their brain after earlier whole-brain radiotherapy (WBRT), and clinical and radiological responses were observed (Lin *et al*, 2008). Subsequently, a larger multicentre phase 2 study involving 242 patients recently reported a 6% CNS objective response rate to lapatinib alone (defined as ⩾50% volumetric reduction of CNS lesion(s) (Lin *et al*, 2009). In addition, a further 21% patients experienced a ⩾20% volumetric reduction in their CNS lesions, of whom many had significant improvement in neurological symptoms. More recently, Boccardo *et al* (2008) described a significant benefit in their review of patients with CNS metastases from HER2-positive breast cancer treated with capecitabine and lapatinib.

The global lapatinib expanded access programme (LEAP) study was a single-arm open-label clinical trial that allowed access to lapatinib in combination with capecitabine for patients with HER2 overexpressing breast cancers, who had previously received anthracycline, taxane and trastuzumab, with the aim of providing clinical benefit while awaiting regulatory approval in individual countries, and further validating safety data observed in EGF100151 in a larger population (Ryan *et al*, 2008). Entry criteria were similar to those in the pivotal trial; in contrast, however, earlier capectabine use was permitted and patients with cerebral metastatic disease could participate, provided they were on a steroid dose of ⩽2 mg per day of dexamethasone (or equivalent) and had a performance status (PS) of 0 to 2.

We have reviewed our experience of using capecitabine in combination with LEAP looking at both efficacy (progression-free survival, PFS) and safety across the entire United Kingdom. In a cohort from the five largest participating sites (Royal Marsden Hospital, Mount Vernon/Hillingdon, Nottingham, Essex and The Christie Hospital), we looked in more detail at efficacy, systemically and in the CNS. Tumour response rate, TTP and treatment-related toxicities were assessed and compared with those reported in EGF100151. In addition, we performed a separate analysis of the cohort of patients with CNS metastases to assess response in this group.

## Materials and methods

### Study design

This was a single-arm open-label expanded access study of lapatinib in combination with capecitabine for male and female patients with HER2 overexpressing locally advanced breast cancer (LABC) defined as stage IIIb/c with a T4 lesion or metastatic (stage IV) disease. The primary objective was to provide clinical benefit to patients who were not eligible for other ongoing lapatinib clinical trials, with the secondary objective being to evaluate serious adverse events (SAEs) associated with this combination therapy in this population of Her2-overexpressing metastatic and LABC. In total, 4283 patients were recruited worldwide with 356 (8.3%) coming from the United Kingdom; across the five sites in this report, 162 patients were recruited, of whom 34 had evidence of CNS disease. Entry criteria differed from those of EGF100151, in that patients were eligible for LEAP regardless of any previous capecitabine use, presence of CNS involvement or absence of measurable disease. Major inclusion criteria were as follows: adults >18 years with disease progression as defined by Response Evaluation Criteria in Solid Tumors (RECIST) after previous treatment with anthracyclines, taxanes and trastuzumab for HER2 overexpressing LABC or MBC; ECOG PS 0 to 2; life expectancy >8 weeks; adequate haematological, renal and hepatic function; and adequate cardiac function as defined by multigated acquisition scan (MUGA) or echocardiogram (ECHO). Patients with CNS disease were allowed to participate in the study, provided they were asymptomatic and on ⩽2 mg dexamethasone (or equivalent) per day. Exclusion criteria included symptomatic angina, congestive cardiac failure, arrythmias, malabsorption or other gastrointestinal disease affecting absorption of oral medications, pregnancy or lactation and uncontrolled infection. The study was sponsored by GlaxoSmithKline, and received approval from MREC and local hospital R&D departments.

### Treatments

Doses were capecitabine 2000 mg m^–2^ per day in two divided doses for 14 days, followed by a 7-day rest and lapatinib 1250 mg once daily continuously. Treatment continued until disease progression, unacceptable toxicity or patient withdrawal from the study. Dose reductions of up to 50% of the planned lapatinib dose, and dose reductions or delay of the planned capecitabine dose, were permitted according to the severity and recurrence of toxicity experienced.

### Assessments

Patients were assessed for response every 6 weeks for the first 6 months of study, then every 12 weeks, or at a frequency after the study site institution's standard. Data were collected from patient case notes, electronic patient records and case report forms across the five participating sites. Duration of treatment, number of cycles given, previous treatment with capecitabine, sites of metastatic disease, number of lines of previous therapy and main site of disease progression at entry were all documented. Best response to treatment as assessed by the investigator was recorded, and patients were deemed to have had a response if they had a complete response (CR) or partial response (PR) and ‘clinical benefit’ if there had been CR/PR or stable disease (SD) for >6 months. Response was assessed by RECIST criteria as per study protocol. TTP was defined as the time from initiation of study medication until the earliest date of disease progression as assessed by the investigator. Patients with CNS disease were included in the main analysis and also assessed independently.

### Safety

Patients were assessed for safety at each site with clinical and laboratory evaluations every 3 weeks and cardiac monitoring, either MUGA or ECHO, every 12 weeks as per protocol. At the time of data cutoff, 4283 subjects were enrolled in the programme, including 356 subjects from the United Kingdom whose data will be reviewed below. The global safety data have been reported separately (Capri *et al*, 2009) and we have provided a summary of key safety data for the UK patients.

### Statistics

Response was compared between groups by means of Kendalls tau-b. TTP was illustrated by Kaplan–Meier curves and patients were analysed separately according to previous capecitabine exposure. Differences between groups in objective tumour response rate (ORR) with and without previous capecitabine were assessed by means of the log-rank statistic.

## Results

### Patients and treatment

Between February 2007 and June 2008, 356 UK patients were recruited across 27 sites, accounting for 8.3% of the global LEAP population. For our efficacy analysis, 162 patients were enrolled across five major UK sites (Table 1). All patients had been previously treated with anthracyclines, taxanes and trastuzumab, as per protocol; 62 of the 162 (38%) patients had received capecitabine for MBC previously. Patients had received 0–5+ lines of previous treatment in the advanced setting (median of 2.7 lines), with 71 (43.8%) patients having received ⩾3 previous lines of treatment. The median age of our cohort was 51 years (range 28–75), and all were women. The median duration of treatment was 4 months (range 0–24), with a median number of six cycles of treatment completed (range 1–27).

### Clinical efficacy

Across the whole UK study population (*n*=356), median PFS was 21 weeks (95% CI: 17.6–24.7) and median OS was 40.4 weeks (95% CI: 34.7–44.1). In the efficacy cohort (*n*=162), median PFS was 22 weeks (95% CI: 17–27), which was comparable with both the overall UK and global study data (PFS 21.1 weeks, 95% CI: 20.1–22.3). Best tumour response was recorded as assessed by the investigator, according to standard RECIST criteria in those with measurable disease, and clinically in those with skin or chest wall-only disease. In all, 157 of the 162 patients had a formal response assessment, with 21% (95% CI: 15–27%) of patients documented as having an objective tumour response (CR/PR), and 50% (95% CI: 42–58%) deemed to have had clinical benefit that included SD ⩾6 months (Table 2). The five other patients progressed or died before the first 6-week response assessment. There was a significant difference in both ORR and clinical benefit rate (CBR) between those who had received capecitabine previously and those who had not (Table 2), with greater efficacy being observed in patients who were capecitabine naive (ORR 23 *vs* 16.3%, *P*=0.008).

**Figure 1:**
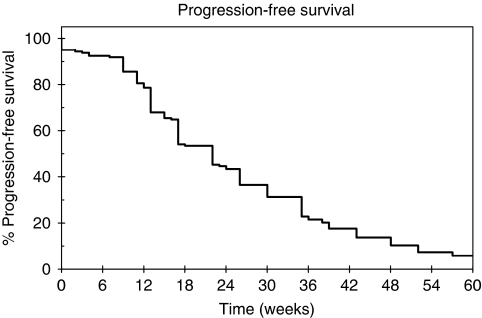
Time to progression illustrated by a Kaplan–Meier Curve for the 162 patients assessed for response.

**Figure 2:**
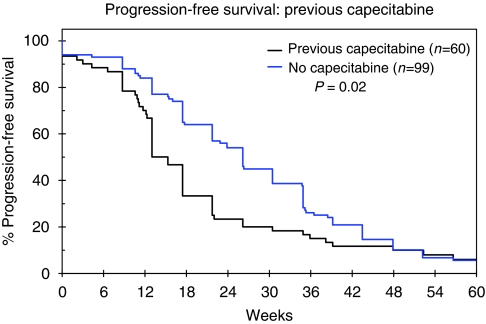
Time to progression by previous capecitabine exposure, illustrated by Kaplan–Meier Curves for the cohort of patients assessed for response.

TTP was recorded in weeks and illustrated by means of a Kaplan–Meier curve (Figure 1). Median TTP was 22 weeks (95% CI: 17–27) and differed according to previous capecitabine exposure, with patients who had received prior capecitabine having a poorer TTP than those patients who had not; median TTP was 15 weeks (95% CI: 10–21) compared with 26 weeks (95% CI: 20–32) *P*=0.02 (Figure 2).

### Patients with CNS disease

Of the cohort of 162 patients, 34 had documented CNS involvement. The median age among this subgroup of patients was younger (49 years, range 28–64 years) compared with that of the overall LEAP study population, which had a median age of 51 years (range 28–75 years) Patients had a similar number of lines of previous treatment (mean 2.4, range 1–5+) compared with the overall study population (mean 2.5) (Table 3). In 23 of the 34 patients, the brain was the predominant site of disease at entry to the study; however, only 17 out of 34 (50%) had overt progression in the brain and/or neurological symptoms at entry, other patients having more significant systemic progression with largely stable brain disease. Most patients (94%) had received WBRT between 1 and >12 m before entry into LEAP, one patient had previously undergone neurosurgery alone, and two patients received no previous CNS loco-regional treatment. The median duration of treatment was 5 months (range 1–15) and a median number of seven cycles of treatment were completed (range 2–22).

**Figure 3:**
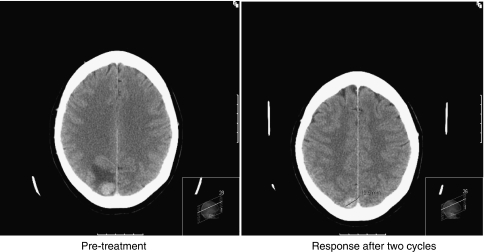
Response of CNS metastases to lapatinib and capecitabine in the absence of previous whole-brain radiotherapy. This 61-year-old woman with HER2-positive breast cancer was diagnosed in 2002. Subsequently, she received three lines of trastuzumab-containing chemotherapy for metastatic breast cancer (paclitaxel, vinorelbine and capecitabine). In July 2007, she developed headache, nausea and vomiting. CT brain revealed three metastases with associated cerebral oedema. She required only a very small dose of steroid and was entered into the LEAP study without previous local therapy (neither neurosurgery nor WBRT) and a combination of capecitabine and lapatinib was her first treatment for CNS disease. She had an excellent clinical response after two cycles, with complete resolution of her headache, nausea and intermittent vomiting, as well as radiological improvement on repeat CT scan with resolution of cerebral oedema and volume reduction of metastases.

**Figure 4:**
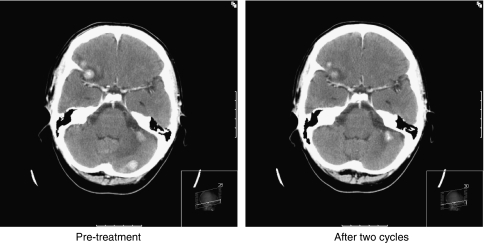
Case study 2 and scan pictures illustrating response. This 47-year-old woman was diagnosed in 1995 with HER2-positive primary breast cancer. In June 2005, she developed metastatic disease (bones, liver and lung) and was treated with eight cycles of docetaxel, trastuzumab and pamidronate to which she had a good response, continuing on maintenance trastuzumab and pamidronate with stable disease. In August 2006, she developed headache and left hand tremor, and a CT scan of her brain confirmed multiple cerebral metastases that were treated with whole-brain radiotherapy, whereas her systemic disease remained stable on continued trastuzumab and pamidronate. In February 2007, she developed increasing headache, and a CT scan confirmed CNS disease progression. In March 2007, she commenced capecitabine and lapatinib in the LEAP study, and, within 6 weeks, a repeat CT scan showed a significant reduction in tumour volume. Her CNS disease remained stable for a further 14 cycles of treatment before progressing in March 2008.

Best tumour response in CNS metastases was assessed and recorded by the investigator, according to RECIST, and 33 (97%) patients had a formal response assessment by CT or MRI imaging. In total, 7 out of 33 (21% 95% CI: 9–39%) patients were documented as having an objective response (one CR, six PRs), and 16 out of 33 (48% 95% CI: 31–60%) were deemed to have had clinical benefit (Table 3). One patient died before the first 6-week response assessment occurred. The ORR in CNS was lower in those previously treated with capecitabine, with 2 out of 12 patients (16.7%) responding compared with 6 out of 20 patients (30%) in the capecitabine-naive group (*P*=0.2); one patient had unknown previous capecitabine exposure. Two illustrative case histories showing the effects of lapatinib and capecitabine in CNS metastases are described in Figures 3 and 4.

**Figure 5:**
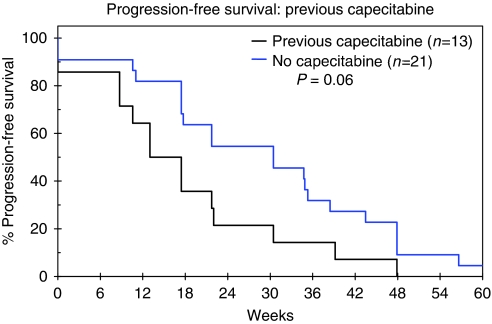
Time to progression for the subgroup of patients with brain metastases (*n*=34), according to previous capecitabine exposure, illustrated by Kaplan–Meier Curves, from the cohort of 162 patients assessed for response.

For the 34 patients with CNS metastases, most of whom had progressed despite previous radiotherapy, the median TTP was 22 weeks (95% CI: 15–28). The numbers were too small to be statistically significant, but again response varied with capecitabine exposure. Median TTP for those previously treated with capecitabine was 17 weeks (95% CI: 13–22) compared with 30 weeks (95% CI: 15–45) for the capecitabine-naive group (*P*=0.06) (Figure 5).

### Safety data

In the UK patients (*n*=356), as of 30 September 2008, a total of 163 SAEs were reported from 89 (25%) subjects. The most frequently reported SAE was diarrhoea with 18 reports, 94% of which were assessed as treatment related. Overall, 42% (69 out of 163) of the SAEs reported were assessed as drug related by the investigator. Diarrhoea, vomiting, nausea, dehydration and palmar-plantar erythrodysaesthesia syndrome are included in the core safety information for lapatinib. A summary of the most frequently reported SAEs can be found in Table 4.

**Figure 6:**
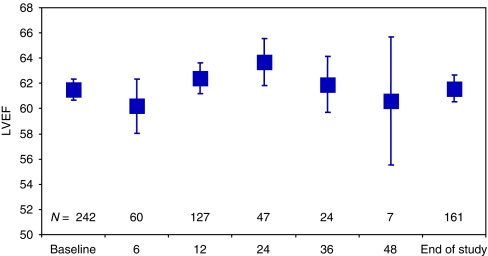
Changes in LVEF from the overall UK Safety data (*n*=356) (graph shows the mean LVEF and 95% CI at each time point.).

Left ventricular ejection fraction (LVEF) was evaluated using MUGA scans or echocardiogram during lapatinib phase I, II and III trials. In the UK study population, there was no significant change in LVEF during treatment (Figure 6). Two subjects in the United Kingdom experienced a decreased ejection fraction as defined by the protocol-specific serious definition (a drop of >20% from baseline and below the institution's lower limit of normal). They experienced a drop in LVEF of 35% and 38.5% from baseline at 120 and 48 days, respectively, after starting treatment. In both cases, the decrease was considered to be possibly related to treatment with laptinib and capecitabine.

No pulmonary events in the United Kingdom met the study criteria for SAE reporting and withdrawal (pulmonary symptoms that are NCI CTC Grade 3 or greater). Ten serious haepatobiliary events (defined as ALT>8xULN, ALT>5 × ULN for >2/52, ALT>3 × ULN with clinical signs or symptoms of hepatitis or ALT>3 × ULN Bilirubin>2 × ULN with >35% direct) were reported in the United Kingdom: one event was considered to be possibly associated with lapatinib and capecitabine administration, three serious haepatobiliary events were associated with disease progression and were not thought to be related to lapatinib and capecitabine administration, and five of the remaining six events were associated with disease progression and seriousness may have been due to an event other than the haepatobiliary event.

## Discussion

The overall efficacy and safety of the UK LEAP cohort of 356 patients matches the findings of the recently published Global LEAP study of 4283 patients (Capri *et al*, 2009). Within our more detailed group of 162 UK patients, we documented an ORR to lapatinib and capecitabine of 21%, and a clinical benefit of 50% in a heavily pre-treated population of women with HER2-positive advanced breast cancer. This compares very favourably with the pivotal EGF100151 study, in which earlier treatment with capecitabine was not permitted, and ORRs of 22% were reported for lapatinib and capecitabine compared with 14% in the capecitabine alone group, with a CBR of 27% and 18%, respectively (Geyer *et al*, 2006).

The cohort of UK patients assessed for response in this report showed a median TTP of 22 weeks, although it should be noted that these patients differed from the EGF100151 population in that they were more heavily pre-treated. In particular the UK cohort included patients with CNS metastases and 38.3% had received capecitabine previously. We showed a significant difference in TTP in our patients between those who had received prior capecitabine and those who had not (15 *vs* 26 weeks, *P*=0.02), with a similar benefit and trend towards significance in the subset with CNS disease (17 *vs* 30 weeks, *P*=0.06). This is again comparable with the global data from the LEAP/ATU analysis in which PFS was 18.4 weeks (95% CI: 17.9–19.4) for those receiving capecitabine earlier compared with 23.9 weeks (95% CI: 22.3–25) for those who were capecitabine naive. Indeed, the TTP of 26 weeks that we report for patients who were capecitabine naive is very similar to the investigator-assessed median TTP of 23.6 weeks (5.9 months) reported in EGF100151 (Geyer *et al*, 2006).

Safety in the overall UK population was again comparable with that of EGF100151, with no significant differences in LVEF being documented over the study period in either case. Two patients (*n*=356) in the UK LEAP study developed a decrease in LVEF and five patients were reported as having an ‘asymptomatic cardiac event’ in EGF100151, four of whom were in the combination therapy group.

It is well documented that patients with HER2 overexpressing tumours seem to have a higher incidence of intracranial metastases (up to 30%) (Clayton *et al*, 2004), and the brain is often described as a ‘sanctuary site’ because of difficulties with cytotoxic agents or monoclonal antibodies such as trastuzumab crossing the ‘blood–brain barrier’. There are anecdotal reports of responses to capecitabine monotherapy in CNS metastases in breast cancer (Ekenel *et al*, 2007). More recently, there have been further reports of responses to lapatinib-based therapy. After an initial single-centre phase II study of lapatinib monotherapy in patients with HER2-positive breast cancer who developed progressive CNS metastases after prior loco-regional therapy (Lin *et al*, 2008), a larger multicentre phase II study involving 242 patients reported a 6% CNS ORR to lapatinib alone (Lin *et al*, 2009). In an exploratory analysis, a further 21% patients experienced a ⩾20% volumetric reduction in CNS lesions, with many having significant improvement in neurological symptoms. An association was observed between volumetric reduction and improvement in PFS and neurological signs and symptoms. Subsequently, within this study, a cohort of 50 patients whose CNS disease progressed on lapatinib monotherapy entered an extension phase involving treatment with both capecitabine and lapatinib, with an ORR of 20%, with 40% patients having a volumetric reduction of ⩾20% in their CNS lesions.

In a previous report of 138 patients with brain metastases treated with lapatinib and capecitibine within the LEAP study and the French ATU programme, Boccardo *et al* (2008) reported an ORR of 18% (CR and PR), with a further 47% of patients achieving SD, although the duration of SD was not recorded. They too noted an improvement in neurological symptoms in 25% of patients. Their patient population had a previous exposure to capecitabine that was similar to ours, with 42% having received it before the study. Thus, our subgroup of 34 patients with CNS metastases who had progressed after previous WBRT compares favourably with these previous studies, with an investigator-assessed response rate of 21% and evidence of clinical benefit in half of the treated patients. As the two case histories illustrate (Figures 3 and 4), the oral regimen of lapatinib and capecitabine can improve neurological symptoms and cause CNS tumours to shrink, with disease control in some patients achieved for up to a year. For patients with symptomatic CNS disease progressing after earlier WBRT, prognosis is normally very poor (weeks to a few months at most); hence, this level of benefit in a hitherto ‘difficult-to-treat’ scenario represents meaningful clinical efficacy.

In EGF100151, although the numbers were small, fewer patients in the combination arm developed CNS metastases compared with those on capecitabine monotherapy (4 *vs* 13), suggesting that lapatinib may also have an effect in prevention. Lapatinib, being a small molecule TKI, seems to penetrate more readily into the CNS, as illustrated by pre-clinical data that showed *in vitro* inhibition of phosphorylation of EGFR, HER2, downstream signalling proteins and cell proliferation in brain-seeking breast cancer cell lines MDA-MB-231-BR (with and without HER2), and a corresponding 50–53% reduction in the number of brain metastases developed by nude mice injected with these cells when treated with lapatinib, compared with a control group (Gril *et al*, 2008). It will be interesting to observe whether outcomes from the adjuvant ALTTO trial differ between arms of the study in the numbers of patients developing CNS metastases at relapse. If lapatinib proves to be not only an effective treatment for HER2-positive breast cancer but also helps reduce the likelihood of developing CNS metastases when given in an adjuvant setting, it will have a significant impact on the course of this disease.

In conclusion, we consider these data to be important in supporting the use of lapatinib in combination with capecitabine in Her2+ MBC, particularly in patients who are capecitabine naive and in those who have progressive and symptomatic CNS metastases following earlier WBRT. There is now increasing evidence to suggest that this is an effective and reasonably well-tolerated treatment for patients who progress in CNS as well as in non-CNS sites after previous treatment with trastuzumab-containing regimens. Given the overall improved outlook and better survival for patients with HER2-positive breast cancer, there is a real need for further targeted therapies once patients have become trastuzumab resistant, and in particular, for those with CNS disease for whom treatment options are even more limited.

## Figures and Tables

**Table 1:** Patient characteristics and previous treatments given for the cohort of 162 patients from the five centres studied

Patients		162
		
Centre	RMH	47
	Mount Vernon	27
	Nottingham	38
	Essex	21
	Christie Hospital	29
		
Age	Median (range)	51 (28–75)
		
Main site of disease	Local/soft tissue only	33
	Bone	9
	Visceral (liver/lung/mediastinum)	97
	Brain^a^	23
		
Prior chemotherapy	Anthracycline/taxane/trastuzumab	162 (100%)
	*Previous capecitabine*	
	Yes	62 (38.3%)
	No	99
	N/A	1
		
Number of previous	0	5
lines of treatment	1	29
	2	56
	3	44
	4	13
	5+	14
	Not recorded	1

a There were 34 patients in total with central nervous system disease, but only 23 had progression in the brain or the brain as the main site of metastatic disease at entry into the trial.

**Table 2:** Duration of and response to treatment overall and according to prior capecitabine exposure for the 162 patients assessed for response

Best tumour response	CR	2 (1.2%)
	PR	31 (19.7%)
	SD	90 (57.3%)
	PD	31 (19.7%)
	NA/not recorded	5
		
Objective response rate (CR and PR)	33/157	21% (95% CI: 15–27%)
		
Clinical benefit rate (CR, PR and SD>6/12)	78/157	50% (95% CI: 42–58%)
		
Best tumour response according to previous capecitabine exposure	Yes (*n*=62)	No (*n*=99)
		
CR	1 (1.6%)	1 (1%)
PR	9 (14.5%)	22 (22%)
SD	28 (45.1%)	61 (61.6%)
PD	21 (33.9%)	13 (13%)
N/A	3	2

Abbreviations: CR=complete response; PD=progressive disease; PR=partial response; SD=stable disease.

**Table 3:** Treatment centres, patient characteristics and best response to treatment for the subgroup of patients with brain metastases from the 162 patients assessed for response

**Patients with brain mets**		
Patients		34
		
Centre	RMH	14
	Mount Vernon	4
	Nottingham	4
	Essex	3
	Christie	9
		
Age	Median (range)	49 (28–64)
		
Number of lines chemotherapy	1	5
treatment for MBC pre-trial	2	15
	3	11
	4	1
	5+	1
		
	Mean number	2.4
		
		
Best response	CR	1 (3%)
	PR	6 (18.2%)
	SD	19 (57.6%)
	PD	6 (18.2%)
	N/A or not recorded	1
		
Objective response rate (CR/PR)	7/33	21% (95% CI: 9–39%)
		
Clinical benefit rate (CR/PR/SD>6 months)	16/33	48% (95% CI: 31–60%)
		

Abbreviations: CR=complete response; MBC=metastatic breast cancer; PD=progressive disease; PR=partial response; SD=stable disease.

**Table 4:** Most frequently reported drug-related SAEs in the United Kingdom

**MedDRA PT**	**Drug-related SAEs**	**% (*n*=356)**
*Diarrhoea*	17	4.8
*Vomiting*	11	3
*Nausea*	6	1.7
*Dehydration*	4	1.3
Chest pain	3	0.8
Pyrexia	3	0.8
Neutropenia	3	0.8
Dyspnoea	2	0.56
Pulmonary embolism	2	0.56
Fatigue	2	0.56
Cellulitis	2	0.56
Mucosal inflammation	1	0.28
*Palmar–plantar erythrodysaesthesia syndrome*	1	0.28

Abbreviations: SAEs=serious adverse events.

Italicized events are listed in the core safety information for lapatinib.

## Appendix

Dr R Agrawal, Cancer Clinical Trials Unit, Oncology Department of Royal Shrewsbury Hospital, Mytton Oak Road, Shrewsbury SY3 8XQ, UK.

Dr P Barrett-Lee, Velindre Cancer Centre Clinical Trials Unit, Velindre Road, Whitchurch, Cardiff CF14 2TL, UK.

Dr K Benstead, 3 Counties Cancer Research Network, The Chapel, St Paul's Medical Centre, Swindon Road, Cheltenham GL50 4DP, UK.

Dr J Bishop, Clinical Trials Unit, North Wales Cancer Treatment Centre, Bodelwyddan, Denbighshire LL18 5UJ, UK.

Dr D Bloomfield, Rosaz Cottage, The Royal Sussex County Hospital, Eastern Road, Brighton, East Sussex BN2 5BE, UK.

Dr A Bowman, Edinburgh Cancer Centre, Western General Hospital, Crewe Road South, Edinburgh EH4 2XU, UK.

Dr M Brunt, Cancer Clinical Trials Unit, University Hospital of North Staffordshire, Princes Road, Stoke-on-Trent ST4 7LN, UK.

Dr S Chan, Oncology Research Centre, Nottingham University Hospital, Hucknall Road, Nottingham NG5 1PB, UK.

Professor N Davidson, Oncology Research, Ground Floor, West Wing 2, Broomfield Hospital, Court Road, Chelmsford, Essex CM1 7ET, UK.

Dr S Dean, Poole Hospital NHS Trust, Dorset Cancer Centre, Longfleet Road, Poole BH15 2JB, UK.

Dr J Dent, Oncology Research Department, The Learning Centre, Huddersfield Royal Infirmary, Acre Street, Lindley, Huddersfield HD3 3EA, UK.

Dr P Dyson, Oncology Department, Cumberland Infirmary, Newtown Road, Carlisle CA2 7HY, UK.

Dr P Ellis, Oncology Department, 4th Floor Thomas Guy House, Guy's Hospital, London SE1 9RT, UK.

Dr C Gallagher, Barts and The London Hospital, London EC1A 7BE, UK.

Dr T Gulliford, Oncology Research Office, Room 20, Exton 1, St Mary's Hospital, Milton Road, Portsmouth PO3 6AD, UK.

Dr T Hickish, Oncology Department, Royal Bournemouth Hospital, Castle Lane East, Bournemouth, Dorset, UK.

Dr S Johnston, Department of Medicine – Breast Unit, The Royal Marsden NHS Foundation Trust, Fulham Road, London SW3 6JJ, UK.

Dr A Jones, Oncology Department, Royal Free Hospital, Pond Street, London NW3 2QG, UK.

Dr D Miles, Academic Oncology Unit, Mount Vernon Hospital, Rickmansworth Road, Northwood HA6 2RN, UK.

Dr P Murray, Clinical Trials Dept, Essex County Hospital, Lexden Road, Colchester CO3 3NB, UK.

Dr K McAdam, Peterborough District Hospital, Thorpe Road, Peterborough PE3 6DA, UK.

Dr S McAleer, N. Ireland Cancer Clinical Trials Unit, East Podium, C-Floor, Belfast City Hospital, Lisburn Road, Belfast BT9 7AB, UK.

Dr C Price, Clinical Trials Unit, Bristol Haematology & Oncology Centre, Horfield Road, Bristol BS2 8ED, UK.

Dr J Stebbing, Department of Medical Oncology, 1st Floor, E Wing, Charing Cross Hospital, Fulham Palace Road, London W6 8RF, UK.

Dr R Stein, Cancer Clinical Trials Unit, 1st Floor Central, 250 Euston Road, London NW1 2PQ, UK.

Dr A Wardley, Breast Cancer Research Team, The Christie Hospital NHS Trust, Wilmslow Road, Withington, Manchester M20 4BX, UK.

Dr C Wilson, Cambridge Cancer Trials Centre, Addenbrookes Hospital, Hills Road, Cambridge CB2 0QQ, UK.

## References

[bib1] Bendell JC, Domchek SM, Burstein HJ, Harris L, Younger J, Kuter I 2003) Central nervous system metastases in women who received trastuzumab based therapy for metastatic breast carcinoma. Cancer 97(12): 2972–2977 12784331 10.1002/cncr.11436

[bib2] Boccardo F, Kauffman B, Baselga J, Dieras V, Link J, Casey M, Fittipaldo A, Oliva C, Zembryki D, Rubin S (2008) Evaluation of lapatinib plus capecitabine in patients with brain metastases from Her2+ breast cancer enrolled in the expanded access program (LEAP) and French authorisation temporaire d’utilisation (ATU). J Clin Oncol (Meeting Abstracts) 26: 64s (Abstr 1094)

[bib3] Cameron D, Casey M, Press M, Lindquist D, Pienkowski T, Romieu CG, Chan S, Jagiello-Gruszfeld A, Kaufman B, Crown J, Chan A, Campone M, Viens P, Davidson N, Gorbounova V, Raats JI, Skarlos D, Newstat B, Roychowdhury D, Paoletti P, Oliva C, Rubin S, Stein S, Geyer CE (2008) A Phase III randomized comparison of lapatinib plus capecitabine versus capecitabine alone in women with advanced breast cancer that has progressed on trastuzumab: updated efficacy and biomarker analysis. Breast Cancer Res Treat 112(3): 533–543 18188694 10.1007/s10549-007-9885-0

[bib4] Capri G, Jerusalem G, Chang J, Jiang Z, Link J, Chen SC, Ro JS, De Placido S, Conte PF, Kaufman B, Johnston S, Schütte HJ, Cwiertka K, Preston A, Selzer M, Rosenlund J, Zembryki D, Oliva C, Parikh R (2009) An open-label expanded access study of lapatinib and capecitabine in patients with ErbB2-overexpressing locally advanced or metastatic breast cancer. Ann Oncol; E-pub ahead of print 2 November 2009. Advance access. doi:10.1093/annonc/mdp373 10.1093/annonc/mdp373 19815649

[bib5] Clayton AJ, Danson S, Jolly S, Ryder WD, Burt PA, Stewart AL, Wilkinson PM, Welch RS, Magee B, Wilson G, Howell A, Wardley AM (2004) Incidence of cerebral metastases in patients treated with trastuzumab for metastatic breast cancer. Br J Cancer 91: 639–643 15266327 10.1038/sj.bjc.6601970 PMC2364775

[bib6] Ekenel M, Hormigo AM, Peak S, Deangelis LM, Abrey LE (2007) Capecitabine therapy of central nervous system metastases from breast cancer. J Neurooncol 85(2): 223–227 17611719 10.1007/s11060-007-9409-0

[bib7] Geyer CE, Forster J, Lindquist D, Chan S, Romieu CG, Pienkowski T, Jagiello-Gruszfeld A, Crown J, Chan A, Kaufman B, Skarlos D, Campone M, Davidson N, Berger M, Oliva C, Rubin SD, Stein S, Cameron D (2006) Lapatinib plus capecitabine for Her2-positive advanced breast cancer. N Engl J Med 355: 2733–2743 17192538 10.1056/NEJMoa064320

[bib8] Gril B, Palmieri D, Bronder JL, Herring JM, Vega-Valle E, Feigenbaum L, Liewehr DJ, Steinberg SM, Merino MJ, Rubin SD, Steeg PS (2008) Effect of lapatinib on the outgrowth of metastatic breast cancer cells to the brain. J Natl Cancer Inst 100(15): 1092–1103 18664652 10.1093/jnci/djn216 PMC2575427

[bib9] Lin NU, Carey LA, Liu MC, Younger J, Come SE, Ewend M, Harris GJ, Bullitt E, Van den Abbeele AD, Henson JW, Li X, Gelman R, Burstein HJ, Kasparian E, Kirsch DG, Crawford A, Hochberg F, Winer EP (2008) Phase II trial of lapatinib for brain metastases in patients with human epidermal growth factor receptor 2-positive breast cancer. J Clin Oncol 26(12): 1993–1999 18421051 10.1200/JCO.2007.12.3588 PMC4524351

[bib10] Lin NU, Diéras V, Paul D, Lossignol D, Christodoulou C, Stemmler HJ, Roché H, Liu MC, Greil R, Ciruelos E, Loibl S, Gori S, Wardley A, Yardley D, Brufsky A, Blum JL, Rubin SD, Dharan B, Steplewski K, Zembryki D, Oliva C, Roychowdhury D, Paoletti P, Winer EP (2009) Multicenter phase II study of lapatinib in patients with brain metastases from HER2-positive breast cancer. Clin Cancer Res 15(4)): 1452–1459 19228746 10.1158/1078-0432.CCR-08-1080

[bib11] Meric F, Hung MC, Hortobagyi GN, Hunt KK (2002) Her2/neu in the management of invasive breast cancer. J Am Coll Surg 194(4): 488–501 11949754 10.1016/s1072-7515(02)01121-3

[bib12] Nicholson RI, Gee JMW, Harper ME (2001) EGFR & cancer prognosis. Eur J Cancer 37(Suppl 4): S9–S15. 8 11597399 10.1016/s0959-8049(01)00231-3

[bib13] Ryan Q, Ibrahim A, Cohen MH, Johnson J, Ko CW, Sridhara R, Justice R, Pazdur R (2008) FDA drug approval summary: lapatinib in combination with capecitabine for previously treated metastatic breast cancer that overexpresses HER-2. Oncologist 13(10): 1114–1119 18849320 10.1634/theoncologist.2008-0816

[bib14] Slamon DJ, Leyland-Jones B, Shak S, Fuchs H, Paton V, Bajamonde A, Fleming T, Eiermann W, Wolter J, Pegram M, Baselga J, Norton L (2001) Use of chemotherapy plus a monoclonal antibody against Her2 for metastatic breast cancer that overexpresses Her2. N Engl J Med 344(11): 783–792 11248153 10.1056/NEJM200103153441101

